# Learning to Obtain Reward, but Not Avoid Punishment, Is Affected by Presence of PTSD Symptoms in Male Veterans: Empirical Data and Computational Model

**DOI:** 10.1371/journal.pone.0072508

**Published:** 2013-08-27

**Authors:** Catherine E. Myers, Ahmed A. Moustafa, Jony Sheynin, Kirsten M. VanMeenen, Mark W. Gilbertson, Scott P. Orr, Kevin D. Beck, Kevin C. H. Pang, Richard J. Servatius

**Affiliations:** 1 Department of Veterans Affairs, VA New Jersey Health Care System, East Orange, New Jersey, United States of America; 2 Stress & Motivated Behavior Institute, Department of Neurology and Neurosciences, New Jersey Medical School, Rutgers, The State University of New Jersey, Newark, New Jersey, United States of America; 3 Department of Psychology, Rutgers, The State University of New Jersey, Newark, New Jersey, United States of America; 4 Graduate School of Biomedical Sciences, Rutgers, The State University of New Jersey, Newark, New Jersey, United States of America; 5 Marcs Institute for Brain and Behaviour & School of Social Sciences and Psychology, University of Western Sydney, Sydney, Australia; 6 Department of Veterans Affairs, Manchester, New Hampshire, United States of America; 7 Harvard Medical School and Massachusetts General Hospital, Boston, Massachusetts, United States of America; Centre national de la recherche scientifique, France

## Abstract

Post-traumatic stress disorder (PTSD) symptoms include behavioral avoidance which is acquired and tends to increase with time. This avoidance may represent a general learning bias; indeed, individuals with PTSD are often faster than controls on acquiring conditioned responses based on physiologically-aversive feedback. However, it is not clear whether this learning bias extends to cognitive feedback, or to learning from both reward and punishment. Here, male veterans with self-reported current, severe PTSD symptoms (PTSS group) or with few or no PTSD symptoms (control group) completed a probabilistic classification task that included both reward-based and punishment-based trials, where feedback could take the form of reward, punishment, or an ambiguous “no-feedback” outcome that could signal either successful avoidance of punishment or failure to obtain reward. The PTSS group outperformed the control group in total points obtained; the PTSS group specifically performed better than the control group on reward-based trials, with no difference on punishment-based trials. To better understand possible mechanisms underlying observed performance, we used a reinforcement learning model of the task, and applied maximum likelihood estimation techniques to derive estimated parameters describing individual participants’ behavior. Estimations of the reinforcement value of the no-feedback outcome were significantly greater in the control group than the PTSS group, suggesting that the control group was more likely to value this outcome as positively reinforcing (i.e., signaling successful avoidance of punishment). This is consistent with the control group’s generally poorer performance on reward trials, where reward feedback was to be obtained in preference to the no-feedback outcome. Differences in the interpretation of ambiguous feedback may contribute to the facilitated reinforcement learning often observed in PTSD patients, and may in turn provide new insight into how pathological behaviors are acquired and maintained in PTSD.

## Introduction

In the wake of exposure to a traumatic event, some individuals develop post-traumatic stress disorder (PTSD), which includes re-experiencing, avoidance, emotional numbing, and hyperarousal symptoms. In some populations, prevalence can be quite high. For example, a recent study [1] found that three to four months after return from combat in Iraq, 18% of Army recruits and 20% of Marines met diagnostic criteria for PTSD, while a recent re-examination of data on Vietnam-era veterans found a lifetime PTSD prevalence of 19% [2]. In addition, a large percentage of trauma victims who do not meet full symptom criteria for PTSD diagnosis present with a number of PTSD symptoms; such subclinical or subthreshold PTSD may cause significant distress and functional impairment [3], [4], [5], [6]. In both clinical and non-clinical groups, arousal symptoms may appear earlier than, and predict the emergence of, other symptom categories [7], while avoidance and re-experiencing symptoms may follow a more linear trajectory [8].

The fact that symptom presence and severity may increase over time following exposure to a traumatic event suggests that PTSD reflects, at least partially, an ongoing process whereby pathological responses are learned and maintained. An influential model of PTSD assumes that some PTSD symptoms reflect classically-conditioned associations; specifically, initially-neutral cues (conditioned stimuli, CS) that are present at the time of exposure to a traumatic event (unconditioned stimuli, US) become associated with the strong emotional responses (unconditioned response, UR) generated by the traumatic event. Through association with the US, these CSs acquire the ability to evoke conditioned emotional responses (CR) that may be similar in form to the UR (e.g., fear) [9], [10]. Thus, PTSD may be characterized by rapid acquisition of a CR, and/or slow extinction of the CR when the CS is no longer paired with the US. This raises the question of why some individuals form such strong, extinction-resistant associations while others, exposed to comparably severe traumatic events, do not. One possible explanation is that some individuals are generally more prone to acquire CRs in the first place, and these individuals would be therefore more likely to develop symptoms. If so, then these individuals should show facilitated acquisition of associative learning not limited to learning about cues present during a traumatic event. Consistent with this idea, a number of studies have now documented facilitated acquisition of classically-conditioned responses in individuals with PTSD symptoms [11], [12], [13], [14], although other studies have reported impairment [15] or no effect of PTSD symptoms [16], [17], [18]. It is also not yet clear whether the facilitated learning often observed in PTSD applies equally to learning involving reward (i.e. learning how to obtain positive outcomes) and punishment (i.e. avoiding or escaping from aversive outcomes).

In the current study, male veterans self-assessed for current, severe PTSD symptoms (PTSS) were tested on a probabilistic classification task [19] that interleaves reward learning and punishment learning. On each trial, participants view a stimulus and are asked to categorize it. For some stimuli, correct classification results in a reward (point gain) and incorrect classification results in no feedback; for other stimuli, incorrect categorization results in a punishment (point loss) and correct categorization results in no feedback. Thus, individuals’ performance on reward and punishment trials can be directly contrasted, as can individuals’ interpretation of the ambiguous “no-feedback” outcome, which can signal either failure to obtain reward or successful avoidance of punishment.

Prior studies with this and similar tasks interleaving reward and punishment trials have reported group differences; for example, damage to or manipulation of brain dopamine systems selectively affects learning to obtain reward but not learning to avoid punishment [19], [20], [21] while damage to anterior insula or dorsal striatum selectively impairs punishment learning but has no effect on reward learning [22]. Serotonergic manipulations have variously been found to selectively enhance the ability to predict punishment with no effect on the ability to predict reward [23], or to impair behavioral and neural representations of reward but not punishment [24], or to affect both reward and punishment learning [25]. Therefore, in the current study, we investigated first whether veterans with PTSS would show facilitated learning based on cognitive feedback, compared to a control group of veterans with few or no PTSD symptoms, and second whether this would apply equally to reward-based and punishment-based trials.

In addition, even among healthy controls, there may be individual differences in relative rates of reward and punishment learning. Specifically, reward learning has been shown to correlate with the personality trait of novelty seeking and punishment learning with the personality trait of harm avoidance [19]. Another personality trait of interest is behavioral inhibition (BI), a tendency to withdraw from or avoid novel social and non-social stimuli, which confers vulnerability to PTSD and anxiety disorders [26], [27], [28]. Among young adults (college students), those with BI are faster to acquire both reward and punishment learning, compared to uninhibited peers [29]. Since BI is also high among veterans with PTSS [30], we expected that there might similarly be facilitated reward and punishment learning in veterans with BI.

Finally, any observed differences in reward or punishment learning between control and PTSS veterans could arise from a number of potential mechanisms, including differences in learning from positive vs. negative feedback, whether the ambiguous “no-feedback” outcome is interpreted as failure to obtain reward or as successful avoidance of punishment, and the tendency to continue making previously-rewarded responses rather than exploring new responses. One way to investigate the degree to which such factors may influence individual participants’ behavior is through the use of computational models.

Reinforcement learning (RL) models of decision making [31], [32] assume that the learner links situations to actions by trial-and-error learning. Eventually, the learner chooses actions that are expected to maximize reward and/or minimize punishment. Prediction error (PE), the difference between expected and experienced outcomes, is used to update the learner’s expectations and guide action selection. PE is positive when there is unexpected reward (or if a predicted punishment fails to materialize), and negative when there is unexpected punishment (or omission of expected reward). A large body of single-unit neurophysiology studies implicates phasic dopamine signals in encoding PE during classical and instrumental conditioning [33], [34], [35], while human functional neuroimaging studies have revealed activity consistent with PE in several brain areas including the striatum, a target of dopamine neurons [36], [37], [38], [39], [40]. In tasks that consider reward and punishment learning separately, it has been demonstrated that activity in anterior striatum correlates with reward-based but not punishment-based PE estimates [41], while damage to the anterior insula and degeneration of dorsal striatum each selectively impair punishment but not reward learning [22]. Together, these studies suggest that different brain systems may be involved in calculating and responding to PE during reward and punishment learning.

To capture this dissociation, we used a version of the gain-loss model [20], [42], [43], which includes separate learning rate parameters α_G_ and α_L_ to update the model following trials with a better-than-expected outcome (positive PE) or following trials with a worse-than-expected outcome (negative PE), respectively. Another parameter governing choice behavior in the model is*β*, an “inverse gain” parameter that reflects the tendency to repeat previously successful responses or explore new ones. In addition, we considered *R0*, the reinforcement value of the no-feedback outcome, which was allowed to range between the values of explicit reward (+1) and punishment (–1) to capture the fact that different people might weight this outcome as truly neutral (*R0* near 0) or as representing either a successfully avoided punishment (similar to a reward) or a missed opportunity for reward (similar to a punishment). For each individual, parameter values were identified that caused the model to display behavior that best mimicked an individual’s observed behavior, in order to examine whether differences in these parameters might suggest mechanisms underlying different performance in PTSS and control groups.

## Methods

### Ethics Statement

This study was approved by the VA NJHCS Institutional Review Board and conducted in accordance with the Declaration of Helsinki. All participants provided written informed consent before initiation of any experimental procedures, and were compensated $40 for their participation in a two-hour session.

### Participants

Ninety-six male veterans were initially recruited from the VA New Jersey Health Care System (NJHCS), East Orange, NJ. One participant’s data were lost due to computer failure, and eight participants were later excluded (described further below), leaving a final sample of *N* = 87, with a mean age of 53.2 years (SD 9.2) and education of 14.0 years (SD 2.0). Sixty-nine veterans self-identified race as black or African American, 12 as white, and 6 as Mixed Race or Other; 6 self-identified ethnicity as Hispanic. When asked to report conflicts in which they had served, 34 reported Vietnam, 7 reported Gulf War (Operations Desert Storm/Shield), 6 reported Operations Enduring Freedom/Iraqi Freedom (OEF/OIF), 10 reported other conflicts (3 Granada, 2 Kosovo/Bosnia, 3 Lebanon/Beirut, 2 Panama), and 31 reported no specific conflict or peacetime service. (Numbers add to greater than 87 due to one participant who reported service in multiple conflicts.)

Participants were tested in two cohorts (*n* = 45 in the first and *n* = 42 in the second). There were no differences for any recorded variable between the two cohorts (independent-samples *t*-tests, all *p*>0.200), and so data from the two cohorts were pooled for subsequent analysis.

Participants were divided into two groups, those self-reporting current severe PTSD symptoms (PTSS group), and a control group. For inclusion in the PTSS group, participants were required to score at least 50 on the PTSD Checklist-Military version (PCL-M) [44], a 17-item self-report questionnaire that assesses presence and frequency of PTSD symptoms in response to stressful military experiences; symptoms are rated according to how much they have bothered the participant in the past month. PCL-M scores of 50+, indicative of current, severe levels of PTSD symptoms, have been shown to be a predictor of PTSD in military samples [44], [45]. In the current sample, 48 participants (55.2%) met this criterion score and were included in the PTSS group.

For inclusion in the control group, participants were required to score below 50 on the PCL-M and also to be free of current antidepressant medication, since antidepressant medication is mainly based on serotonergic modulation, which has been implicated in reinforcement learning [23], [24], [25]. 39 participants met these criteria and formed the control group. An additional 8 veterans were tested who scored below criterion on the PCL-M, but who declined to provide information regarding current medication use (n = 2) or who reported treatment with antidepressants for conditions other than PTSD (n = 6); data from these eight veterans were not included in the analysis. Within the final set of n = 39 in the control group, 11 reported using other psychoactive medication, such as sleep aids or pain medications.

Because antidepressant medication is common among PTSD patients, and because assignment to medical treatment cannot be expected to be random, use of antidepressants was not treated as an exclusion criterion for the PTSS group. However, we did conduct secondary analyses to determine whether performance differed among those self-reporting current antidepressant use (n = 23), other psychoactive medication (again, typically sleep aids or pain medications; n = 14), or no psychoactive medication (n = 11).

### Questionnaires

In addition to the PCL-M all participants completed the Adult and Retrospective Measures of Behavioural Inhibition (AMBI/RMBI) [46], and the Combat Exposure Scale (CES) [47], since both the personality trait of BI and history of exposure to combat have been identified as risk factors for development of PTSD in veterans [48], [49], [50], [51] and may modify expression of symptoms.

The AMBI is a 16-item self-report inventory that assesses current (adult) BI. AMBI scores have been shown to correlate with measures of anxiety proneness [46], [52] and with PTSD symptom severity [11], [30]. The RMBI is a similar 18-item inventory that assesses childhood (retrospective) BI. As originally published, the RMBI included a “do not remember” option for each question, in which case the response for that question was undefined; we used a modified version of the RMBI that eliminates the “do not remember” response option [11], [30]. Following published cutoff scores [46], participants scoring ≥16 on the AMBI and ≥12 on the RMBI were classified as consistently inhibited; those scoring <16 on the AMBI and <12 on the RMBI were classified as consistently uninhibited; and the remainder were classified as inconsistent.

The CES is a 7-item self-report questionnaire that assesses exposure to stressful military events. Items are rated for frequency, duration, and degree of exposure; total CES score is calculated by summing weighted item ratings. Following prior studies [11], [53], veterans with a CES score of 0-7 were classified as non-combat while those scoring ≥8 were classified as having a history of exposure to combat.

### Behavioral Task

The probabilistic classification task was administered on a Macintosh iMac or equivalent computer, programmed using the SuperCard language (Solutions Etcetera, Pollock Pines, CA). The task took about 20 minutes to complete. A cardboard mask was used to cover the keyboard except for two labeled keys that participants could press to enter their responses. At the start of the experiment, participants received instructions about the task and two practice trials, one involving reward feedback and one involving punishment feedback (see Text S1 for details of instruction and practice trials).

**Figure 1:**
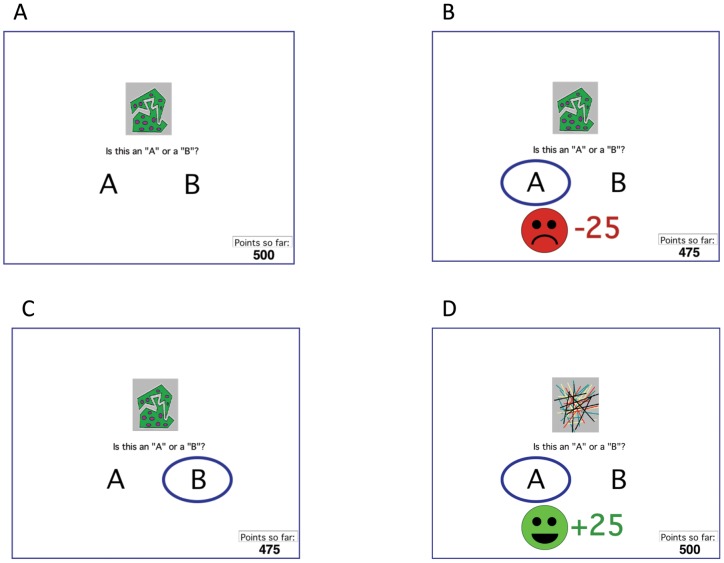
Example screen events from the behavioral task. (A) On each trial, the participant sees a stimulus and is asked to categorize that stimulus as “A” or “B”. The chosen category is circled, and corrective feedback may appear. For some stimuli (punishment trials), incorrect classification is punished with point loss (B) while correct classification receives no feedback (C); for other stimuli (reward trials), correct classification is rewarded with point gain (D) while incorrect classification receives no feedback. The task is probabilistic, so a stimulus does not belong to the same category on every trial (refer Table 1).

Trial events followed those previously published [19]. On each trial, the participant saw a stimulus and was asked to categorize it as belonging to category “A” or “B” using the labeled keys (Figure 1A). For each participant, four stimuli were randomly assigned to be S1, S2, S3, and S4. The task was probabilistic, meaning that stimuli S1 and S3 belonged to category A on 80% of trials and to category B on the remaining 20% of trials, while S2 and S4 belonged to category B on 80% of trials and to category A on the remaining 20% of trials (Table 1). S1 and S2 were “reward” stimuli in that correct classification produced feedback and a gain of 25 points, while incorrect classification produced no feedback. S3 and S4 were “punishment” stimuli in that incorrect classification produced feedback and a loss of 25 points, while correct classification produced no feedback (Figure 1B,C,D). Thus, the “no feedback” outcome was ambiguous, as it could signal either omission of reward (on S1 and S2 trials) or successful avoidance of punishment (on S3 and S4 trials). The participant’s point tally was shown at the bottom of the screen and was initialized to 500 points at the start of the experiment. The task contained 160 trials, divided into four blocks containing 10 trials with each stimulus (8 with the common category and 2 with the uncommon category). For each participant, trial order was randomized within a block. On each trial, the computer recorded whether the participant made the optimal categorization (i.e. category A for S1 and S3, or category B for S2 and S4), regardless of the actual outcome on that trial.

**Table 1:** Category and feedback structure of the probabilistic reward and punishment learning task.

Stimulus	Category Membership	Feedback if correct	Feedback if incorrect
**S1**	80% category A, 20% category B	+25 points	No feedback
**S2**	20% category A, 80% category B	+25 points	No feedback
**S3**	80% category A, 20% category B	No feedback	–25 points
**S4**	20% category A, 80% category B	No feedback	–25 points

Data from the probabilistic classification task were scored in terms of percent optimal responding across the 80 punishment trials and the 80 reward trials. In addition, we classed participants as “solvers” if they made at least 65% optimal responding (52 out of 80 trials) on reward or punishment trials (significantly better than chance, binomial test, two-tailed *p*<0.01), or as “non-solvers” if they did not reach this performance criterion on either reward or punishment trials.

### Computational Model

Each participant’s behavior was modeled using a RL model adapted from Frank et al.’s gain-loss model [20]. Specifically, on each trial *t*, a stimulus *s* was presented. Two expectancy values, *Q[A,s]* and *Q[B,s]*, represented the expected outcomes associated with responding to *s* with category A or category B, respectively. All *Q* were initialized to 0 at the start of a simulation run. On each trial *t* = 1..160, given a stimulus *s*, the probability *Pr(A)* of choosing category A was calculated using a softmax function [54]:
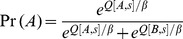



where *ß* was an inverse gain parameter that could range from 0 to 1 and that specified the tendency to choose the response with highest expectancy value (low *ß*) or choose a response at random (high *ß)*.

Next, the model was provided with the same feedback *R* as the participant received on that trial. *R* could take one of three values: *R+* (reward), *R*– (punishment), or *R0* (no feedback). Prediction error *PE* was then computed as *PE = R*–*Q[r,s]* where *r* was the participant’s actual response (category A or B) and *s* was the current stimulus. In the simulations reported here, *R+* was fixed at +1 and *R*– at –1, but *R0* was a free parameter that could vary from –1 (*R*–) to +1 (*R+*).

The expectancy values *Q* were then updated based on whether the outcome *R* was better (*PE*>0) or worse (*PE*<0) than expected given the current stimulus-response pairing (*r,s*):
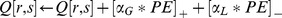



where α_G_ and α_L_ were learning rates associated with gain and loss trials, respectively, and could range independently from 0 to 1.

In summary, the model reported here contained four free parameters: α_G_ and α_L,_ *ß*, and *R0*. Each of these parameters was explored across a range of values (in steps of 0.05 for *ß*, α_G_ and α_L,_ and in steps of 0.1 for *R0*). An alternate model, including a perseveration parameter *P,* was also investigated, but as this did not provide significantly better fit to subject data, the simpler four-parameter model was preferred (see Text S2).

Model fit was assessed by computing log likelihood estimates (LLE) to estimate the *a priori* probability of the data, given a particular combination of parameter values:
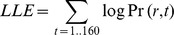



across all 160 trials, where *Pr(r,t)* is the probability that the model makes the same response *r* as the participant on trial *t*. Estimated parameters for each participant were defined as the values of α_G_ and α_L,_ *ß* and *R0* that together resulted in the largest LLE (closest to 0) for that participant’s data.

### Data Analysis

For questionnaire data, we conducted *t*-tests to compare questionnaire scores between control and PTSS groups, and chi-square tests (Yates correction applied for 2×2 tables) to compare differences in case frequency (e.g. combat, BI) between control and PTSS groups. Pearson’s *r* was also used to examine correlations between scores.

For behavioral data, the main analyses were univariate ANOVA on total points and mixed-model ANOVA on percent optimal responding for reward and punishment trials, with factors of PTSD group (PTSS vs. control), BI (inhibited, uninhibited, inconsistent), history of exposure to combat (combat vs. non-combat), and use of psychoactive medications (yes vs. no). Post-hoc univariate ANOVAs or *t*-tests were conducted as needed to further examine significant results. We also conducted correlation analysis (Pearson’s *r*) to examine possible relationships between individual participants’ performance on reward vs. punishment trials, and chi-square tests (with Yates correction for 2×2 tables) to compare proportions of solvers and non-solvers between control and PTSS groups.

For model data, Pearson correlations were used to examine relationships between model fit (LLE), estimated parameters, and behavioral performance. To examine possible relationships between estimated model parameters and PTSD symptoms, the main analyses were mixed-model ANOVAs on the estimated parameters, with factors of PTSS, combat and BI, with post-hoc univariate ANOVA or *t*-test to further examine significant results.

For all statistical analyses, the threshold for significance was set at alpha = 0.05. Bonferonni correction was used to protect against inflated risk of Type I error under multiple comparisons; the corrected alpha is provided in the text when obtained *p*-values were less than 0.05 but greater than the corrected alpha. Test statistics and degrees of freedom are reported both for omnibus tests as well as for any post-hoc tests to investigate interactions identified by the omnibus test; note that, depending on the tests used, the degrees of freedom may differ for the omnibus and post-hoc tests. For ANOVAs including a within-subject factor, the assumption of sphericity was verified using Mauchly’s test, with Greenhouse-Geisser correction if the assumption was violated; for *t*-tests, the assumption of equality of variance was verified using Levene’s test, with Welch’s *t*-test used if the assumption was violated. Note that these corrections adjust the degrees of freedom, which can include fractional parts. For correlation analyses, Spearman’s rho was used instead of Pearson’s *r* where data violated assumptions of linearity. In all figures representing central tendency, error bars indicate SEM; asterisks indicate significant between-group comparisons.

## Results

### Questionnaires

Mean scores for the questionnaires in PTSS and control groups are provided in Table 2. Based on CES scores, 13 veterans (33.3%) in the control group, and 22 veterans (45.8%) in the PTSS group, were classed as having history of exposure to combat; this difference in case frequency between PTSS and control groups was not significant (Yates-corrected χ^2^ = 0.93, *p* = 0.336).

**Table 2:** Mean (and SD) of age and education, and questionnaire scores, in the control and PTSS groups.

	Control Group (*n* = 39; PCL-M<50)	PTSS Group (*n* = 48; PCL-M≥50)
**Age (years)**	52.2 (10.8)	54.0 (8.4)
**Education (years)**	13.9 (2.0)	14.0 (2.0)
**Psychoactive Medication**	28 no; 11 yes (excluding antidepressant)	11 no; 37 yes (including 23 antidepressant)
**Combat Exposure Scale (CES)**	6.0 (7.4)	10.4 (11.0)
**Adult Measure of Behavioural Inhibition (AMBI)**	15.1 (4.5)	21.1 (5.3)*
**Retrospective Measure of Behavioural Inhibition (RMBI)**	11.9 (5.5)	13.9 (6.4)

Asterisk indicates significant difference between PTSS and control groups (*t*-test, *p*<0.001).

AMBI and RMBI scores were strongly correlated (*r* = 0.463, *p*<0.001). There was no difference in AMBI or RMBI scores among veterans with vs. without a history of exposure to combat (both *t*<1.5, both *p*>0.100) but AMBI scores were significantly higher in the PTSS than control group (*t*(85) = 5.60, *p*<0.001), although RMBI scores did not differ between groups (*t*(85) = 1.52, *p* = 0.131). Based on AMBI and RMBI scores, 12 veterans in the control group (30.8%) were classed as inhibited and 14 as uninhibited (35.9%), with the remaining 13 classed as inconsistent (33.3%); in the PTSS group, the rates were 28 inhibited (58.3%), 6 uninhibited (12.5%) and 14 (29.2%) inconsistent. Thus, there were more inhibited participants in the PTSS group than in the control group (χ^2^ = 8.80, *p* = 0.012).

### Behavioral task

**Figure 2:**
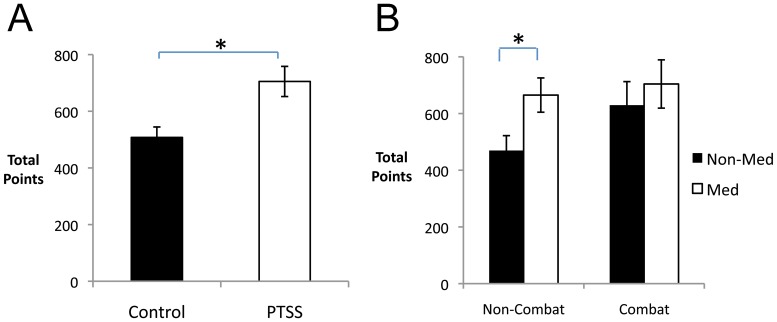
Performance on the behavioral task. (A) Overall, the PTSS group achieved higher total points than the control group (*F*(1,65) = 5.75, *p* = 0.019). (B) There was an interaction of combat history with medication status. Specifically, among those without combat history, those on current psychoactive medications outperformed non-medicated peers (*t*(50) = 2.34, *p* = 0.024).

The PTSS group achieved higher total points on the behavioral task than the control group (Figure 2A; *F*(1,65) = 5.75, *p* = 0.019). There was also an interaction between combat history and use of psychoactive medication (Figure 2B; *F*(1,65) = 6.29, *p* = 0.015); specifically, among veterans with no history of exposure to combat, total points were significantly higher in those taking psychoactive medication (*t*(50) = 2.34, *p* = 0.024) but this effect of medication did not appear in those with a history of exposure to combat (*t*(33) = 0.63, *p* = 0.535). There was also a significant interaction between PTSS and combat history (*F*(1,65) = 4.73, *p* = 0.033), although post-hoc *t*-tests to examine the interaction further found nothing that approached corrected significance levels (all *p*>0.050).

**Figure 3:**
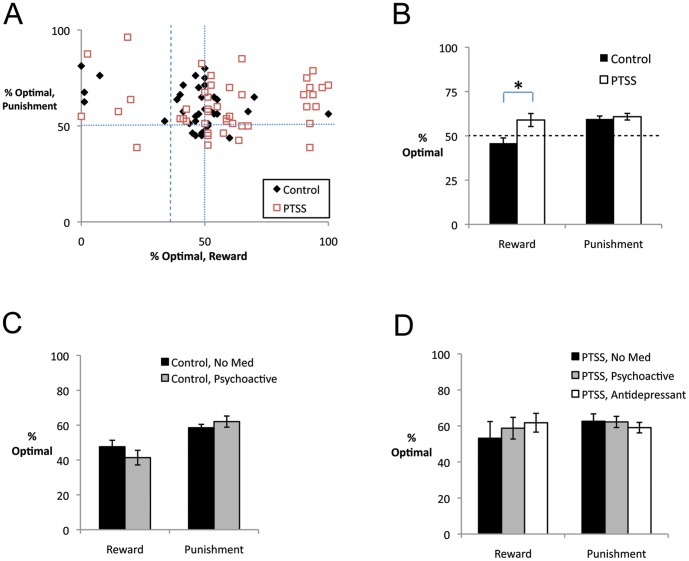
Performance on reward and punishment trials. (A) There was considerable individual variation in performance on reward trials, and no correlation between performance on reward and punishment trials. Vertical and horizontal lines represent chance performance (50%); note 11 participants who made less than 35% optimal responses on reward trials (dashed line) – i.e. reliably chose the *non-optimal* response on reward trials. (B) On reward trials, the PTSS group significantly outperformed the control group (*F*(1,65) = 6.43, *p* = 0.014) but there was no group difference on punishment trials. (C) There was no significant effect of psychoactive medication status in the control group, which specifically excluded participants self-reporting use of antidepressant medications. (D) In the PTSS group, there were no significant differences among those reporting no medication (“No Med”), antidepressant use (“Antidepressant”), or use of psychoactive drugs excluding antidepressants (“Psychoactive”).

Breaking down performance into percent optimal responses on reward and punishment trials revealed considerable individual variation, with no significant correlation between performance on reward and punishment trials (Figure 3A; *r* =  –0.066, *p* = 0.544).

In general, participants performed better on punishment than reward trials (*F*(1,65) = 8.07, *p* = 0.006), and there was again a significant main effect of PTSS (*F*(1,65) = 6.52, *p* = 0.013). The interaction between trial type and PTSS approached significance (*F*(1,65) = 3.66, *p* = 0.060), and there were also interactions between trial type, PTSS, and medication (*F*(1,65) = 4.92, *p* = 0.030), and between trial type, medication, BI, and combat history (*F*(2,65) = 3.52, *p* = 0.035). To further examine these interactions, follow-up univariate ANOVAs were conducted on performance to reward and punishment trials separately. On reward trials, there was a significant effect of PTSS (Figure 3B; *F*(1,65) = 6.43, *p* = 0.014), as well as an interaction between combat and medication (*F*(1,65) = 7.55, *p* = 0.008), although post-hoc tests to explore this interaction did not survive significance (all *p*>0.050). On punishment trials, there was no effect of PTSS, BI, medication or combat history and no interactions (all *p*>0.100). (See also Figure S1 for performance on reward and punishment trials across the experiment, broken down into blocks of 40 trials.)

Finally, the omnibus test revealed additional interactions between medication and combat history (*F*(1,65) = 8.14, *p* = 0.006) and between combat history and PTSS (F(1,65) = 5.30, p = 0.025). Post-hoc repeated-measures ANOVAs to examine these interactions in veterans with no history of exposure to combat revealed only a significant effect of trial type (*F*(1,50) = 7.70, *p* = 0.008) with no effect of medication or PTSS and no interaction (all *p*>0.100); in veterans with a history of exposure to combat, no effects approached significance (all *p*>0.100). Because the PTSS group included both veterans currently using antidepressant medication as well as those reporting other psychoactive medications, supplemental tests were also conducted to examine these subgroups separately; however, no significant differences between medication subgroups were observed (Figure 3C,D; all *p*>0.100).

Because a fairly large number of participants maintained near-chance performance on both the reward and punishment tasks, we also considered the subset of participants who achieved at least 65% optimal responding on the reward or punishment trials. On reward trials, 16 of 48 participants in the PTSS group (33.3%) but only 3 participants in the control group (7.7%) reached this criterion, a significant group difference (Yates-corrected chi-square test, χ^2^ = 6.85, *p* = 0.009). On punishment trials, this group difference was not evident, as 19 participants in the PTSS group (39.6%) and 13 participants in the control group (33.3%) reached criterion (χ^2^ = 0.143, *p* = 0.708).

Defining as “solvers” those participants who met criterion on reward or punishment trials (or both), 15 participants in the control group and 25 in the PTSS group met this criterion. Even in this reduced sample, there remained effects of trial type (*F*(1,38) = 4.09, *p* = 0.050) and of PTSS (*F*(1,38) = 11.53, *p* = 0.002) as well as a type-by-PTSS interaction (*F*(1,38) = 6.24, *p* = 0.017). Specifically, the PTSS group outperformed the control group on reward trials (Figure S2A; *t*(38) = 3.05, *p* = 0.004) but not on punishment trials (*t*(38) = 0.29, *p* = 0.776).

In addition, a group of 11 participants, visible at the left of Figure 3A, performed *below* 35% on reward trials – i.e., picked the non-optimal response on >65% of reward trials. Notice that chance performance is 50%; just as performance >65% represents better-than-chance performance, so performance <35% is significantly *below* what would be predicted if a participant were simply making random responses on these trials. These 11 participants did not differ from the remaining 76 participants on PTSS, BI, combat history or medication status (chi-square tests, all *p*>0.090) or in age, education, AMBI, RMBI or CES scores (*t*-tests, all *p*>0.200). However, despite performing worse than the other participants on reward trials (*t*(85) = 8.76, *p*<0.001), and achieving fewer total points (*t*(85) = 4.47, *p*<0.001), these 11 participants showed somewhat *better* performance on the punishment trials than the remaining participants (poor reward group: *M* = 67.2, SD 16.8; remaining participants: *M* = 59.2, SD 10.86; *t*(85) = 2.11, *p* = .038). Note that no participants performed at or below 35% optimal on the punishment trials (see Figure 3A).

### Computational Model

For each participant’s data, a unique combination of estimated parameter values resulted in maximal LLE (closest to 0) for that participant. Over all participants, LLE averaged –81.06 (SD 24.0).

LLE was correlated with performance on the punishment trials (*r* = 0.587, *p*<0.001) but not reward trials (*r* = 0.081, *p* = 0.455). (See Figure S3 for scatterplots of LLE and performance.) There were no significant differences in LLE as a function of PTSS group, medication status, BI or combat history (ANOVA, all *F*<3.00, all *p*>0.00 except BI (*F*(2,65) = 2.90, *p* = 0.062).

Over all participants, mean estimated values for the free parameters were α_G_ = 0.27 (SD 0.32), α_L_ = 0.23 (SD 0.34), *β* = 0.34 (SD 0.27), and *R0* = 0.36 (SD = 0.55). There were strong negative correlations between α_G_ and α_L_ (Spearman’s *r* = –0.326, p = 0.002) and between α_G_ and *R0* (Spearman’s *r* = –0.454, *p*<0.001), as well as between *R0* and performance on reward trials (Spearman’s *r* = –0.478, *p*<0.001). There was also a strong negative correlation between *β* and performance on punishment trials (Spearman’s *r* = –0.369, *p*<0.001). No other correlations among estimated parameters or between estimated parameters and performance levels approached corrected significance (all *p*>0.050).

**Figure 4:**
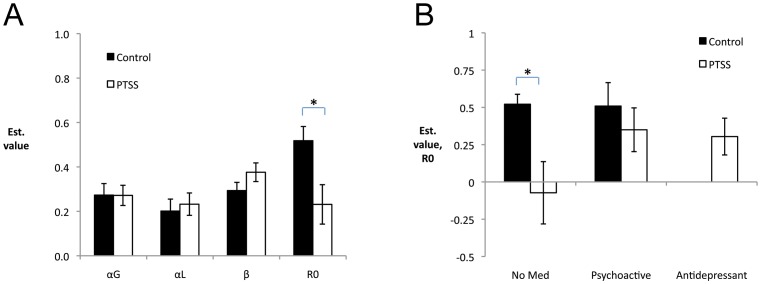
Estimated parameter values, as a function of performance on reward trials. (A) Estimated values for *R0*, the weight of the no-feedback outcome, were significantly larger in the control group than the PTSS group (*t*(81.37) = 2.62, *p* = 0.010), indicating that control participants tended to weight this outcome more similarly to reward. There were no differences between control and PTSS groups in estimated values of the other parameters, α_G_ and α_L_ (learning rates for gain and loss trials) or β (inverse gain parameter). (B) These differences in estimated value of *R0* appeared to be driven primarily by the subset of participants not reporting use of psychoactive medication, with unmedicated participants in the PTSS group having estimated *R0* near 0, significantly less than the value for unmedicated participants in the control group (*t*(37) = 3.56, *p* = 0.001); there was no difference between PTSS participants on antidepressants vs. those on other types of psychoactive medication (*t*(35) = 0.23, *p* = 0.816).

The key modeling question in which we were interested was whether estimated parameters would differ for the PTSS and control groups and, if so, whether this could be used to suggest possible mechanisms underlying the observed group differences. In fact, mixed-model ANOVA on the four estimated parameter values, with factors of PTSS, BI, medication status, and combat history revealed an expected within-subjects effect of parameter (*F*(4,260) = 6.45, *p*<0.001) as well as an interaction between parameter and PTSS group (*F*(4,260) = 3.65, *p* = 0.007). Follow-up *t*-tests on each parameter revealed that the PTSS group had significantly lower estimated values of *R0* than the control group (*t*(81.37) = 2.62, *p* = 0.010); none of the other estimated parameter values differed between groups (all *p*>0.050; Figure 4A). The fact that estimated values of *R0* were larger (farther from 0) in the control group than the PTSS group is consistent with the poorer performance on reward trials by the control group, since more strongly positive values of *R0* would result in the no-feedback outcome being perceived as relatively rewarding, lessening the impact of actual reward (*R+*).

Although the interaction with medication status fell short of significance in the omnibus ANOVA, further analysis revealed that there was a qualitative difference in estimated values of *R0* in unmedicated PTSS participants, compared to other subgroups. Specifically, estimated values of *R0* were close to 0 in the unmedicated PTSS subgroup, while unmedicated controls had estimated values of *R0* that were close to 0.5 (Figure 4B; *t*(37) = 3.56, *p* = 0.001); there was no such difference between PTSS and control participants on psychoactive medication (excluding antidepressants) (*t*(23) = 0.74, *p* = 0.469), and no difference between PTSS participants on antidepressants vs. those on other types of psychoactive medication (*t*(35) = 0.23, *p* = 0.816). No other estimated parameters showed differences as a result of medication status in either PTSS or control groups (all *p*>0.100).

Given that estimated values of *R0* were negatively correlated with performance on reward trials, when the analysis was restricted to “solvers” only, the mean estimated values of *R0* are reduced (Figure S2B). In this restricted sample, there was no longer any significant difference between control and PTSS groups on estimated values of *R0* or any other parameter (all *t*<1.00, all *p*>0.300), although lack of significant group differences must of course be interpreted with caution in a small sample size.

Among just the n = 11 participants who performed significantly below chance (<35% optimal) on reward trials, mean estimated values of *R0* were higher than among the remaining participants (*M* = 0.69, SD = 0.34 vs. remaining participants: *M* = 0.31, SD = 0.55), although the difference fell short of corrected significance (*t*(85) = 2.20, *p* = 0.030). No other differences in estimated parameter values approached corrected significance (all *p*>0.050).

## Discussion

The current study assessed a sample of male veterans with self-reported current, severe PTSD symptoms (PTSS), to determine whether facilitated learning could be observed in a purely cognitive task that involved both learning to obtain reward and learning to avoid punishment. We found that the PTSS group outperformed the control group, in terms of total points won as well as in percentage of participants reaching a performance criterion. The PTSS group showed better performance on reward trials than the control group, with no difference on punishment trials. Using an RL model and maximum likelihood estimation techniques, we found estimated values for the no-feedback outcome (*R0*) that were closer to 0 for the PTSS than control group, suggesting that the PTSS group tended to weight ambiguous feedback as fairly neutral, while the control group tended to weight it as more similar to reward (successful avoidance of possible punishment); this could account for the group difference in performance on reward trials. We discuss each of these points further below.

### Sample characteristics and questionnaire results

In our sample of 87 male veterans, over half met criteria for PTSS. Clinical diagnosis of PTSD requires additional criteria beyond current, severe symptoms, and so it is likely that not all individuals in our PTSS group would satisfy full diagnostic criteria for PTSD. Even so, individuals with subclinical PTSD can display symptoms that may cause significant distress and functional impairment [3], [4], [5], [6].

Several vulnerability factors for PTSD have been identified, such that individuals with these characteristics may be at heightened risk to develop PTSD in the wake of exposure to traumatic events. One such factor is a history of exposure to combat. In the present study, about a third of combat veterans, and about half of non-combat veterans reached criteria for PTSS based on self-report. Thus, there was no evidence that combat history was associated with greater PTSD symptomatology in this sample. Rather, the current data suggest that even veterans without combat exposure can report high rates of PTSD symptoms related to military service. These symptoms could reflect non-combat but service-related stressors including deployment and/or reintegration into civilian life. In fact, a recent study of over 1,500 Marines who deployed in support of conflicts in Iraq and Afghanistan found that deployment-related stressors were even more strongly associated with self-reported PTSD symptoms than was combat exposure [48].

Another vulnerability factor for PTSD is the personality trait of behavioral inhibition (BI). As in our prior studies with samples drawn from this population, we found that self-reported PTSD symptom severity (PCL-M score) was correlated with adult BI [11], [30]. A prior study found better performance on both reward and punishment trials by college undergraduates with high BI compared to their non-inhibited peers [29]. In contrast, in the current study, there was no clear effect of BI on any performance measurement, and no difference in estimated parameters derived by the model among the different BI groups. The difference in results across these two studies may reflect the considerable differences between the subject populations, but may also reflect the fact that, in the current study, the strong effects of PTSS may have masked weaker relationships with BI.

Other vulnerability factors for PTSD exist, which were not assessed in the current study, and which may have additionally contributed to variance in the results. These include (but are not limited to) female gender, childhood trauma, lack of social support, and various genetic and biological factors [55], [56], all of which could be explored in future studies of reward and punishment learning in veterans and other populations with high rates of PTSD symptoms.

### Behavioral task

Overall, the PTSS group outperformed the control group in terms of total points achieved and also in percentage of participants reaching a performance criterion. This finding may sound paradoxical but in fact is generally consistent with a number of other studies showing better associative learning in PTSD patients compared to non-PTSD controls [13], [14], [16], [57], [58], [59], [60], although these prior studies have often used explicitly aversive stimuli such as mild electric shocks [13], [58], airpuffs to the eye [16], trauma-specific pictures [14], or loud noise bursts [60].

Given that the current study used a behavioral task that provided only cognitive feedback (point gain/loss), our findings are consistent with a view that PTSD reflects a general facilitation to acquire associations between stimuli and outcomes. Such facilitated learning would include, but not be limited to, stimuli and outcomes associated with traumatic events. If this facilitation pre-dates onset of PTSD, then it might represent a pre-existing vulnerability that would bias an individual to develop PTSD following exposure to a traumatic event. Alternatively, it is possible that the facilitated learning observed in our PTSS veterans arose only in the wake of exposure to trauma and/or development of PTSD symptoms. Longitudinal studies could be designed to examine these possibilities further.

A previous study that tested categorization learning in participants with and without PTSD, and that also employed purely cognitive feedback, found no learning difference between PTSD and non-PTSD groups, although the PTSD group did show impaired generalization of the acquired associations [61]. However, this prior work did not consider reward and punishment feedback separately, whereas the current study interleaved reward and punishment trials, allowing for the assessment of each. In the current study, the control and PTSS groups performed similarly on trials where the goal was to avoid punishment; however, on trials where the goal was to obtain reward, the PTSS group significantly outperformed controls. This was true even when the sample was restricted only to those participants reaching performance criteria (“solvers”).

The lack of group differences on punishment trials may reflect a ceiling effect, but this seems unlikely since the group average was only about 60% optimal on punishment trials. The observed difference on reward trials can also be interpreted either as a selective facilitation of reward learning in the PTSS group, or as a selective suppression of reward learning in the control group. The first possibility, that the PTSS group showed facilitated reward learning, appears tenable given findings (including those cited above) that PTSD patients often show facilitated learning relative to non-PTSD comparison groups, as well as with a large body of literature documenting that, in general, people weight punishment more strongly than reward [62], which is consistent with the pattern observed in the control group. However, the second possibility, that of suppressed reward learning in the control group, is also tenable, given the relatively low rates of reward learning in this group, compared to other studies with this task where healthy control groups achieved considerably higher performance levels [19], [29], [41], [63]. Given that the control participants in this study were veterans, some of whom had combat exposure, it is conceivable that the control group included individuals who are resilient to PTSD, in the sense of having reduced risk for developing PTSD symptoms in the wake of exposure to traumatic events. This resiliency might express itself as a tendency to interpret neutral or ambiguous feedback as rewarding, which would impair performance on the reward-based trials in the current study, but which might have beneficial effects in everyday life. In fact, related constructs such as positive affect [64] and optimism [65] have been previously suggested as resilience factors that may protect against development of PTSD or promote recovery. Such an interpretation remains speculative based on the current data, and would require longitudinal testing to fully explore; however, the idea seems consistent with the results from the computational modeling, since the control group tended to have estimated values of *R0*>0, weighting ambiguous no-feedback outcomes as more like explicit reward, while the PTSS group tended to have estimated values of *R0* that were closer to zero.

In both the PTSS and control groups, there was considerable individual variability on reward trials, with some participants performing well above chance, some at or near chance, and some well below chance. Specifically, as shown in Figure 3A, a number of participants (including participants from both PTSS and control groups) made fewer than 35% optimal responses on reward trials, meaning that they reliably followed a response rule that involved choosing the non-optimal (seldom-rewarded) response on those trials. The presence of a small number of participants who showed persistent non-optimal responding on reward (but not punishment) trials has also been observed in prior studies with this probabilistic task [19], [63]. Many of these participants performed well on the punishment trials, making it unlikely that non-associative factors such as lack of motivation could completely account for the poor performance on reward trials. Rather, it seems likely that these participants were interpreting the ambiguous “no feedback” outcome as rewarding, in the sense of successful avoidance of punishment, on all trials. There is some evidence that the brain codes omission of expected punishment as similar to reward, and encodes failure to obtain expected reward as similar to punishment [21], [66]. In the context of the current task, those individuals who tended to value the reward and no-feedback outcomes as similar (both indicating successful avoidance of punishment) would have been selectively impaired on the reward trials, since responses leading to explicit reward feedback and responses leading to the no-feedback outcome would have been similarly reinforced. Conversely, this same tendency could potentially facilitate performance on the punishment trials; consistent with this interpretation, the subset of participants who performed well below chance on reward trials actually performed slightly better than the remaining participants on punishment trials.

The lack of correlation between performance on reward and punishment trials (Figure 3A) is also consistent with recent neuroimaging studies that suggest different, possibly opponent, processes for reward and punishment learning. For example, a number of studies have now implicated ventral frontostriatal circuits in encoding reward prediction errors but not necessarily in punishment learning [19], [21], [39], [67], [68], [69]; on the other hand, damage to the anterior insula and degeneration of the dorsal striatum have the opposite effect of impairing the ability to learn to avoid punishment while sparing the ability to learn to obtain reward [22]. Future work could consider functional neuroimaging in veterans and others with PTSD symptoms, to see whether abnormality in those brain areas implicated in reward processing mirror behavioral differences in PTSS and control veterans.

### Computational Model

To further investigate possible mechanisms that might underlie the observed patterns of behavioral performance, we used an RL model to estimate various parameter values for individual participants. The model we used was similar to ones previously used to analyze data from probabilistic learning tasks [41], [70], but was based on the gain-loss model [20], [42], [43] which allows separate learning rates α_G_ and α_L_ for reward and punishment trials, and also included a free parameter *R0* encoding the relative value of the no-feedback outcome, which could range from 0 to *R+* (the reinforcement value of reward) and to *R-* (the reinforcement value of punishment); when *R0* = 0, the value of the no-feedback outcome is truly neutral.

Model fit was evaluated using maximal likelihood estimation procedures to determine the configuration of free parameters that produced the best description (greatest LLE) of the observed data. While some studies have suggested that parameter value estimation for individual participants is highly variable and susceptible to extreme estimations, the practice of identifying a single set of model parameters that best fits all participants’ data [36], [54], [71] may be most appropriate in functional neuroimaging studies where sample size is small and where the primary concern is often estimation of a trial-by-trial prediction error that can be regressed against brain activity. In a study with a larger sample size (*n* = 69), Frank et al. [20] were able to show group differences in estimated parameters for individuals who carried different genetic polymorphisms, and argued that these genetic differences could explain observed dissociations in group behavior. Similarly, in the current study, our larger samples size yielded parameter estimation that was stable enough to observe statistically significant differences between control and PTSS groups.

On average, estimated parameter values for individual participants included positive values of *R0*, meaning that the no-feedback outcome was valued as more similar to reward (+1) than to punishment (–1). As noted above, this would make it somewhat harder to learn the optimal response on reward trials, which require learning to obtain outcome *R+* rather than *R0*, than to learn the optimal response on punishment trials, which require learning to obtain outcome *R0* rather than *R*–. Consistent with this interpretation, there was also a strong negative correlation between individuals’ performance on the reward trials and estimated values of *R0*.

The key question investigated in the modeling work was whether group differences in estimated parameters would occur for control and PTSS groups and, if so, might shed light on possible mechanisms underlying the observed differences in behavior, particularly on reward trials. In fact, estimated values for *R0* were significantly lower (closer to 0) in the PTSS than control group, with no such group differences observed in estimated values for the other free parameters. This is consistent with the behavioral data, in which the PTSS group outperformed the control group on reward (but not punishment) trials, and suggests that participants in the PTSS group were more prone to weight the no-feedback outcome as neutral (and distinct from either reward or punishment), whereas the control group was more likely to weight reward and the no-feedback outcome as similar.

Finally, an interesting interaction emerged from the modeling, specifically, that the group difference in estimated values of *R0* between control and PTSS participants appeared to be driven primarily by the subset in each group who did not report use of psychoactive medication. Among this “non-medicated” subset, estimated values of *R0* were near 0 for the PTSS participants, but close to +0.5 for the controls. By contrast, participants in the PTSS group who self-reported use of antidepressants, or other psychoactive medications, had estimated values of *R0* above 0, which did not differ from the estimated values in unmedicated controls. This suggests the possibility that use of psychoactive medication, including antidepressants, remediates the putatively abnormal weighting of *R0* in PTSS participants. However, since assignment to medication group was not random in this study, nor was it confirmed except through self-report, this conclusion remains tentative.

### Limitations and Future Directions

Several important limitations of the current work suggest additional avenues for future research. First, the current study focused on veterans self-assessed for PTSD symptoms; assessment of PTSD symptoms was made through the PCL-M, a well-normed and well-validated tool that has shown good predictive validity in military samples [44], [45]. However, the PCL-M by design focuses on PTSD symptoms related to stressful military events. As such, assessments based PCL-M scores are not easily translatable to civilian comparison groups. In addition, it is entirely possible that veterans who do not report criterion levels of PTSD symptoms related specifically to military events may nevertheless experience significant PTSD symptoms related to non-military events (e.g. car accidents). Future research could compare rates of PTSD symptoms in veteran and civilian samples, matched for age, education and other demographic variables, and assessed for PTSD symptoms related to both military and non-military events. This would allow further insight into whether the difference in performance by control vs. PTSS groups on reward learning in the current task represents facilitated learning in the PTSS group, or abnormally poor learning among veterans in the control group.

Another limitation of the current work is the focus on male veterans only. The course and expression of PTSD and stress-related symptoms may be different in female veterans [72], [73], [74], [75]. Understanding of how associative learning biases translate into vulnerability will be incomplete without consideration of how gender may modulate these processes.

A final important limitation of the current study is the fairly large number of participants (over 50%) who were classed as non-solvers, meaning that they failed to achieve a relatively lax performance criterion (at least 65% optimal responding on either reward or punishment trials). While it is still true that the PTSS group outperformed the control group on reward learning, even when non-solvers were excluded from analysis, nevertheless future studies could consider simplified variations of this task, where a greater proportion of participants achieve a performance criterion, so see if similar behavioral and modeling results are obtained.

Turning to the modeling, although the RL model produced differences in estimated values of *R0* that could contribute to explaining the group difference in reward learning, nevertheless good model fit is not sufficient to conclude that a given model (and parameters) accurately capture the underlying processes generating the empirically-observed data [76]. Stronger evidence would be if the insights gained from the model can be used to predict future data. One prediction of the current model is that task manipulations that vary the relative value of ambiguous feedback, or the relative strengths of reward and punishment feedback, might affect the pattern of behavior observed in the PTSS vs. control group. This in turn might suggest new therapeutic approaches for behavior modification therapy. Specifically, while current treatment for PTSD-related avoidance symptoms often involves an extinction process (i.e. omission of an expected punisher), it is possible that other strategies, such as differential reinforcement of alternative responses (i.e. provision of rewarding feedback), might prove efficacious.

## Conclusions

The current study observed facilitated learning in veterans with severe PTSD symptoms, in a task that used cognitively-based reward and punishment feedback. This facilitation was specifically attributable to better performance in the PTSS group than control group on reward-based trials. A computational model, applied to individual participant data, was used to estimate several free parameters, including *R0*, the relative reinforcement value of ambiguous no-feedback outcomes. Significantly greater estimated values of *R0* were derived for the control group than for the PTSS group. One interpretation of this finding is that those in the PTSS group interpreted the ambiguous no-feedback outcome as neutral (close to 0) while controls tended to weight it as more similar to reward (i.e., successful avoidance of punishment). This effect appeared to be modulated by presence of psychoactive medication in the PTSS group. Clearly, additional work needs to be done to confirm and extend these findings to other tasks and populations. However, the idea that veterans with severe PTSD symptoms tend to value ambiguous feedback differently than veterans with few or no PTSD symptoms suggests a mechanism that might contribute to the facilitated associative learning often observed in PTSD patients. Gaining a better understanding of how associative learning is altered in PTSD may provide improved insight regarding how pathological behaviors are acquired and maintained in PTSD, which could guide the development of more effective treatments or preventive interventions.

## Supporting Information

Figure S1 **Performance on reward and punishment trials across the course of the experiment**, **broken down into blocks of 40 trials (10 trials with each of the four stimuli).** Mixed-model ANOVA with within-subject factors of trial type (2) and block (4) and between-subject factor of PTSS group revealed a significant effect of trial type (*F*(1,85) = 7.30, *p* = 0.008), a near-significant effect of PTSS (*F*(1,85) = 3.94, *p* = 0.050) and a significant type x PTSS interaction (*F*(1,85) = 3.94, *p* = 0.005). Thus, these data replicate the finding of better performance by the PTSS group than the control group on reward, but not punishment observed in Figure 3B.
(TIF)

Figure S2 **Performance of “Solvers,” defined as participants achieving at least 65% optimal responding on either reward or punishment trials.** (A) Considering only the 15 participants in the control group and 25 in the PTSS group who met this criterion, the PTSS group still outperformed the control group on reward trials (*t*(38) = 3.05, *p* = 0.004) but not punishment trials (*t*(38) = 0.29, *p* = 0.776). (B) However, having removed particularly those participants who performed poorly on reward trials (who tended to have largest estimated values of R0), there is no longer any significant difference between PTSS and control groups on any of the estimated parameters in the model (all *t*<1.00, all *p*>0.300).
(TIF)

Figure S3 **Model fit (LLE) as a function of performance on (A) reward and (B) punishment trials**. LLE was positively correlated with performance (percent optimal responses) on punishment trials (*r* = 0.587, *p*<0.001); this partially reflects the fact that model fit will be greater for participants who demonstrated more deterministic behavior – typically, those performing well will have fairly deterministic response patterns (and correspondingly greater LLE) while those making responses randomly will typically perform poorly (and have correspondingly lower LLE). However, there was not a strong linear relationship on reward trials (*r* = 0.081, *p* = 0.455), primarily due to the subset of participants who performed below 35% optimal on reward trials but who nevertheless performed reasonably well on punishment trials (refer Figure 2A). In fact, when these *n* = 11 participants are excluded from analysis, the remaining 76 participants showed strong positive relationships between LLE and both reward and punishment performance (both *r*≥0.345, both *p*≤0.002); among those *n* = 11 participants themselves, there was a significant positive correlation between LLE and performance on punishment trials (*r* = 0.773, *p* = 0.005) and a negative correlation between LLE and performance on reward trials (*r* = –0.717, *p* = 0.013).
(TIF)

Text S1 **On-screen instructions provided to participants completing the behavioral task.**
(DOCX)

Text S2 **Model fitting experiments.** Expanding the four-parameter model to include a fifth parameter *P* indicating perseveration did not significantly improve the ability of the model to describe the data.
(DOCX)
