# Supplementary figures and images for: Learning to Obtain Reward, but Not Avoid Punishment, Is Affected by Presence of PTSD Symptoms in Male Veterans: Empirical Data and Computational Model
Source: PLoS One. 2013 Aug 27;8(8):e72508. doi: 10.1371/journal.pone.0072508 (PMC3754989; doi:10.1371/journal.pone.0072508)

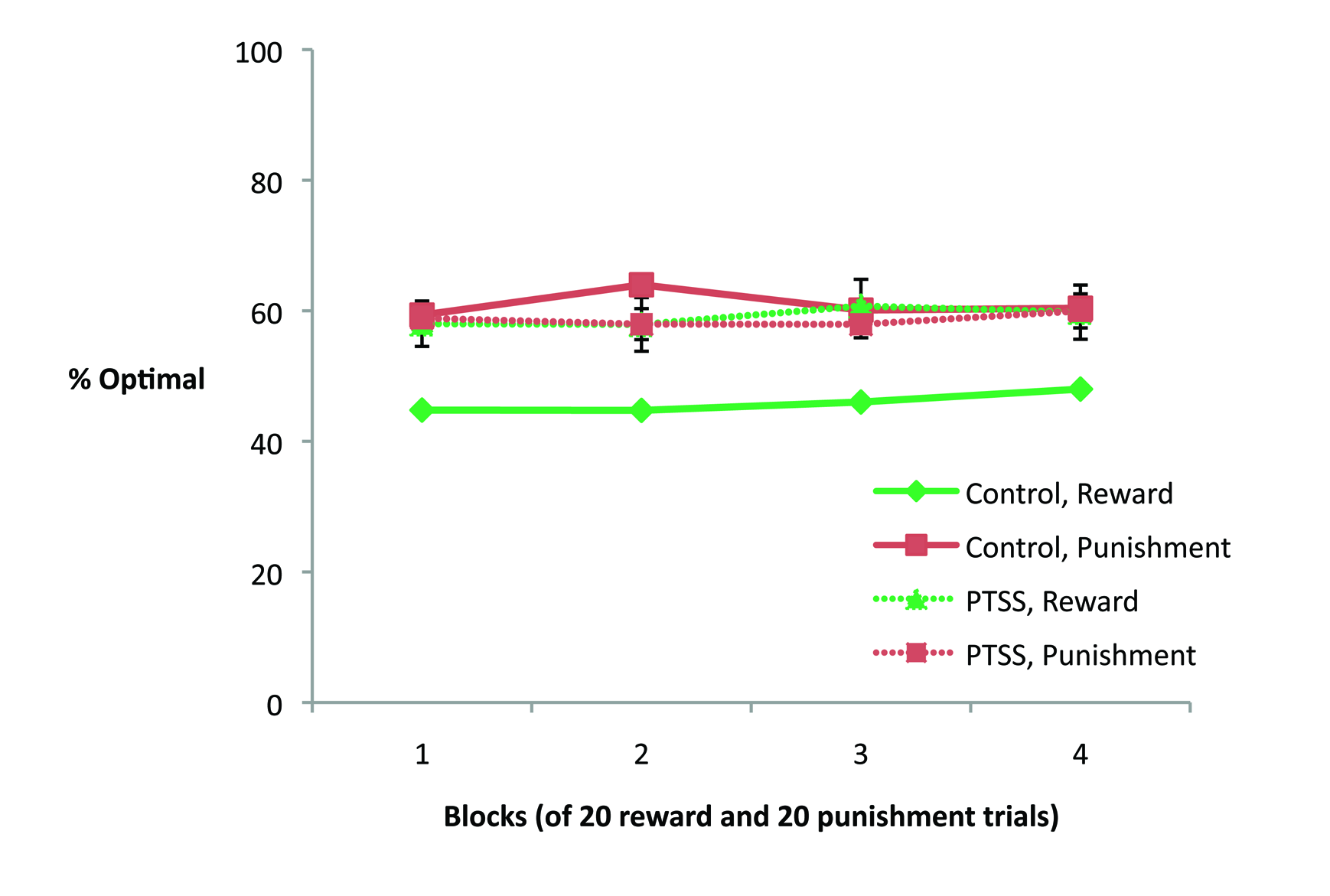

Supplement: Figure S1 — Performance on reward and punishment trials across the course of the experiment, broken down into blocks of 40 trials (10 trials with each of the four stimuli). Mixed-model ANOVA with within-subject factors of trial type (2) and block (4) and between-subject factor of PTSS group revealed a significant effect of trial type (F(1,85) = 7.30, p = 0.008), a near-significant effect of PTSS (F(1,85) = 3.94, p = 0.050) and a significant type x PTSS interaction (F(1,85) = 3.94, p = 0.005). Thus, these data replicate the finding of better performance by the PTSS group than the control group on reward, but not punishment observed in Figure 3B. (TIF) [file pone.0072508.s001.tif]

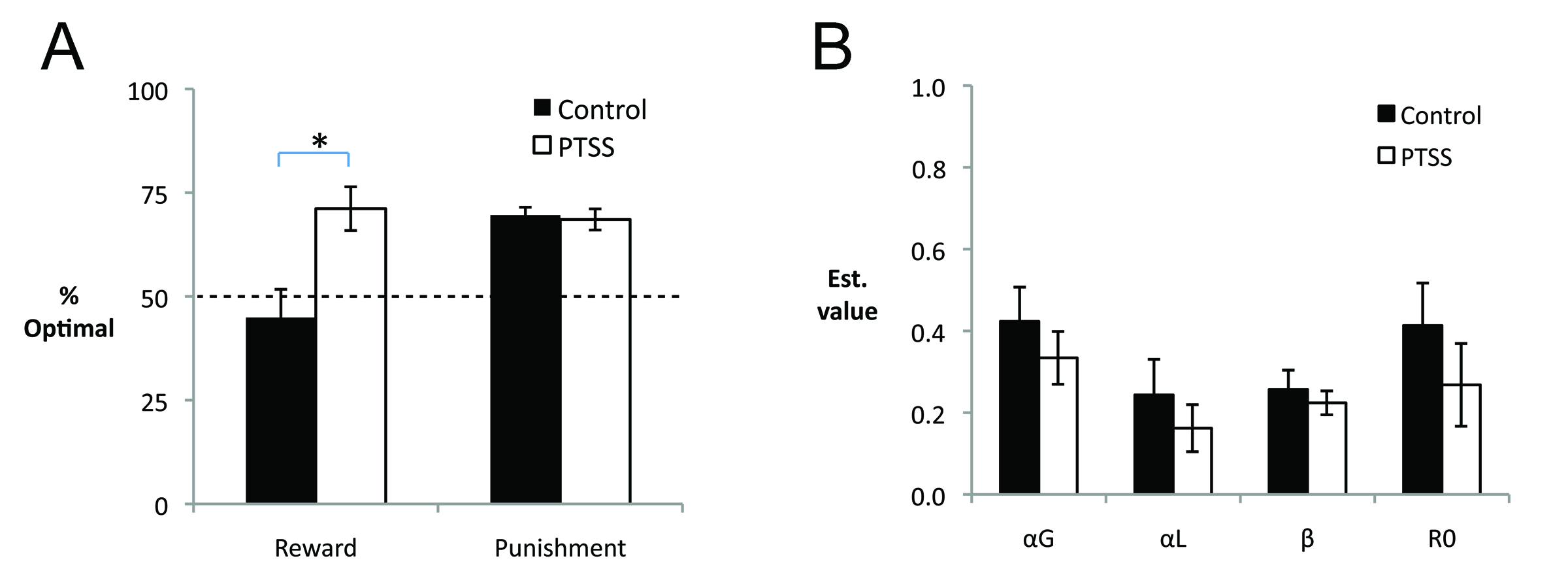

Supplement: Figure S2 — Performance of “Solvers,” defined as participants achieving at least 65% optimal responding on either reward or punishment trials. (A) Considering only the 15 participants in the control group and 25 in the PTSS group who met this criterion, the PTSS group still outperformed the control group on reward trials (t(38) = 3.05, p = 0.004) but not punishment trials (t(38) = 0.29, p = 0.776). (B) However, having removed particularly those participants who performed poorly on reward trials (who tended to have largest estimated values of R0), there is no longer any significant difference between PTSS and control groups on any of the estimated parameters in the model (all t<1.00, all p>0.300). (TIF) [file pone.0072508.s002.tif]

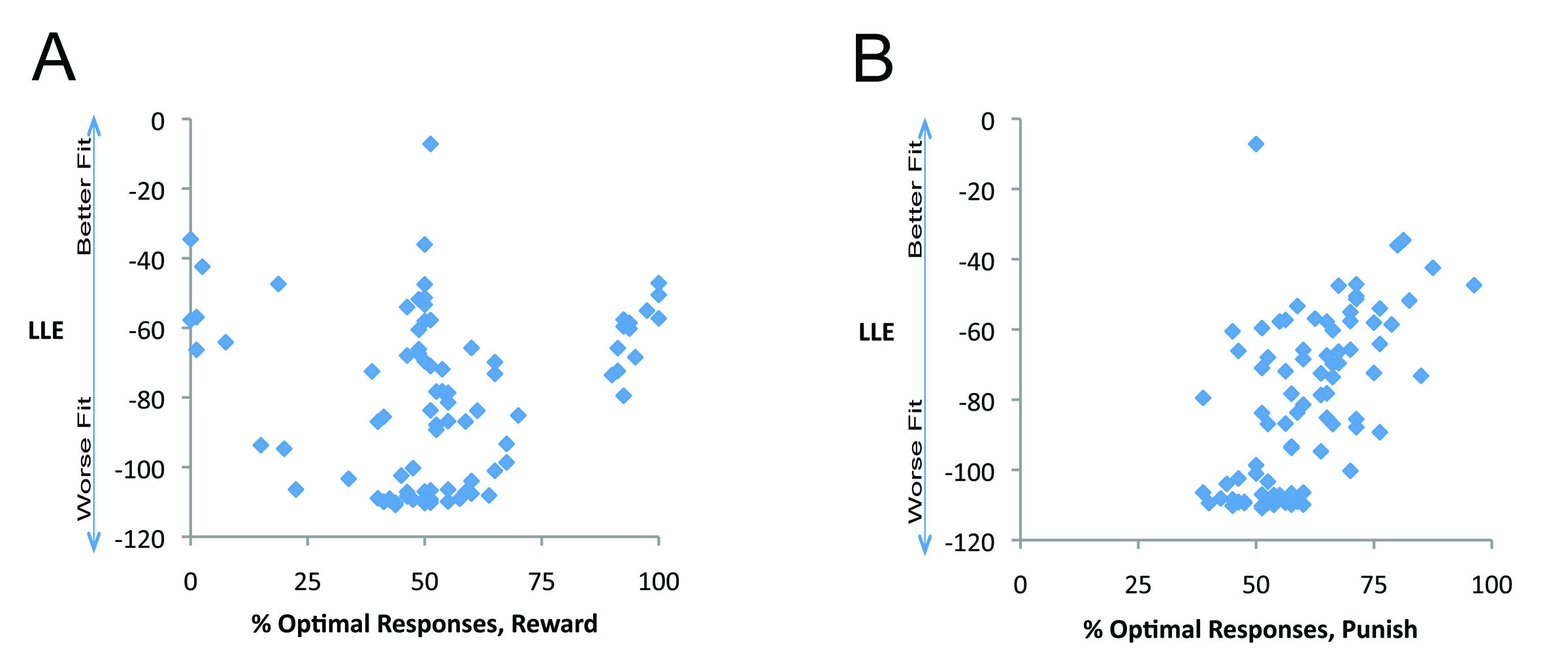

Supplement: Figure S3 — Model fit (LLE) as a function of performance on (A) reward and (B) punishment trials. LLE was positively correlated with performance (percent optimal responses) on punishment trials (r = 0.587, p<0.001); this partially reflects the fact that model fit will be greater for participants who demonstrated more deterministic behavior – typically, those performing well will have fairly deterministic response patterns (and correspondingly greater LLE) while those making responses randomly will typically perform poorly (and have correspondingly lower LLE). However, there was not a strong linear relationship on reward trials (r = 0.081, p = 0.455), primarily due to the subset of participants who performed below 35% optimal on reward trials but who nevertheless performed reasonably well on punishment trials (refer Figure 2A). In fact, when these n = 11 participants are excluded from analysis, the remaining 76 participants showed strong positive relationships between LLE and both reward and punishment performance (both r≥0.345, both p≤0.002); among those n = 11 participants themselves, there was a significant positive correlation between LLE and performance on punishment trials (r = 0.773, p = 0.005) and a negative correlation between LLE and performance on reward trials (r = –0.717, p = 0.013). (TIF) [file pone.0072508.s003.tif]
