# Supplementary material for: Learning to Obtain Reward, but Not Avoid Punishment, Is Affected by Presence of PTSD Symptoms in Male Veterans: Empirical Data and Computational Model
Source: PLoS One. 2013 Aug 27;8(8):e72508. doi: 10.1371/journal.pone.0072508 (PMC3754989; doi:10.1371/journal.pone.0072508)
Supplement: Text S1 — On-screen instructions provided to participants completing the behavioral task. (DOCX) [file pone.0072508.s004.docx]

**Supplementary Text S1: Instructions for the Behavioral Task**

At the start of the behavioral task, participants viewed an instruction screen followed by a series of practice trials to familiarize them with screen events, the possible outcomes (reward, punishment, no feedback), and the probabilistic nature of the task.

Once the keyboard had been covered with a mask, so that only the keys labeled “A” and “B” were available, an instruction screen appeared; the experimenter read these and all subsequent instructions aloud to the participant:

*In this experiment, you will be shown pictures and you will guess whether those pictures belong to category “A” or category “B”. A picture doesn’t always belong to the same category each time you see it. If you guess correctly, you may win points. If you guess wrong, you may lose points. You’ll see a running total of your points as you play. (We’ll start you off with a few points now.) Press the mouse button to begin practice.*

When the participant pressed the mouse button to begin practice, a screen appeared similar to Figure 1A, except that the stimulus was not one of the four serving as S1-S4 in the experiment. The point tally at the bottom right was initialized to 500. Additional instructions appeared at the top of the screen (again, to be read aloud by the experimenter):

*Here is an example. Suppose you think this picture belongs to category “A” – press the “A” key now.*

When the participant pressed the “A” key, the “A” response was circled on the screen, a red unhappy face and “-25” feedback appeared (similar to Figure 1B); the point tally was also decremented from 500 to 475. Instructions appeared at the top of the screen:

*That would have been wrong – and you would have lost some points. But if you had chosen “B” (press the “B” key now)…*

When the participant pressed the “B” key, the “B” response was circled on the screen; there was no other feedback and no change to the point tally. Instructions appeared:

*That would have been correct… and so you wouldn’t have lost any points. Press the mouse button to see another kind of trial.*

When the participant pressed the mouse button, a new screen appeared, similar to that in Figure 1A but with a second practice stimulus. Instructions appeared:

*Here’s another picture. Suppose you think this one belongs to category “B.” Press the “B” key now.*

When the participant pressed the “B” key, the “B” response was circled on the screen, a green smiley face and “+25” feedback appeared, and the point tally was incremented from 475 to 500. Instructions appeared:

*That choice would have been correct – so you would have won some points! But if you had chosen the other key (press the “A” key now)…*

When the participant pressed the “A” key, the “A” response was circled on the screen, with instructions:

*That would have been incorrect – and you wouldn’t have won any points. Click the mouse button to continue.*

When the participant clicked the mouse button, the trial screen disappeared. At this point, the participant would have viewed each possible type of feedback, used each response button twice, and experienced each type of outcome/feedback. A final instruction screen appeared, summarizing the instructions:

*So, for some pictures, if you guess CORRECTLY, you WIN points (but, if you guess incorrectly, you win nothing). For other pictures, if you guess INCORRECTLY, you LOSE points (but, if you guess correctly, you lose nothing). Your job is to win all the points you can – and lose as few as you can. Remember that the same picture doesn’t always belong to the same category. Press the mouse button to begin the experiment.*

Following the mouse click, the experiment proceeded to trials 1-160 as described in the main text.
