# Supplementary material for: Learning to Obtain Reward, but Not Avoid Punishment, Is Affected by Presence of PTSD Symptoms in Male Veterans: Empirical Data and Computational Model
Source: PLoS One. 2013 Aug 27;8(8):e72508. doi: 10.1371/journal.pone.0072508 (PMC3754989; doi:10.1371/journal.pone.0072508)
Supplement: Text S2 — Model fitting experiments. Expanding the four-parameter model to include a fifth parameter P indicating perseveration did not significantly improve the ability of the model to describe the data. (DOCX) [file pone.0072508.s005.docx]

**Supplementary Text S2: Model Fitting**

In building a model to describe behavior, the ideal is to obtain the best, most parsimonious description of the data: i.e. highest LLE with the lowest number of free parameters (*k)*. The computational model reported in the main text included four free parameters α_G_ and α_L,_ *ß*, and *R0*. Whereas the first three parameters encode a tendency to learn from or repeat actions based on prior reinforcement, some prior models [1,2] have in addition considered a perseveration parameter, *P*, that encodes a tendency to repeat prior or avoid actions (regardless of reinforcement). High values of *P* are associated with strong perseveration, whereas low values of *P* suggest spontaneous alternation. To incorporate this tendency, the response rule becomes:

**

where *c[r,s]* is a working memory trace that holds a record of the last response *r* to stimulus *s*. The working memory traces are initialized to 0, and updated after each trial as *c[r,s]🡨 1* for the current *r* and *s*; for all other response and actions *c[r,s]🡨 c[r,s]*d*, where *d* is a decay parameter (here, *d*=0.95). For the simulations reported in the main text, *P*=0.

However, we also explored whether better model fit could be obtained by allowing *P* to vary as a free parameter. **Supplemental** **Table S1** compares the average estimated parameters, and degree of fit (LLE) for the standard four-parameter model as reported in the main text, as well as for a five-parameter model where *P* varied as a free parameter from -5 to +5 in steps of 0.1. In assessing model fit while taking model complexity into account, we used the Bayesian information criterion (BIC), defined as *BIC*= *k*ln(n)-*2**LLE*, where *n* is the number of observations (sample size); low values of BIC indicate better, more parsimonious fit [3]. As shown in Table S1, BIC did not differ between the two models (paired-samples *t*-test, *t*(92)=0.29, *p*=0.775). Another metric for comparing model fit is the χ^2^-of-change as more free parameters are added, defined as -2*LLE_A_*-(-2*LLE_B_*), where model B is a subset of model A (some free parameters in model A are held fixed in model B) [4]. Given this definition, there was again no significant difference between models (χ^2^=-4.69, p>0.050). In other words, by both metrics, the more complex model including *P* did not produce significantly better descriptions of the data. Accordingly, in the results reported in the main text, we present the simpler model with only four free parameters.

**References Cited**

1. Daw ND, Doya K (2006) The computational neurobiology of learning and reward. Current Opinion in Neurobiology 16: 199-204.

2. Schönberg T, Daw ND, Joel D, O'Doherty JP (2007) Reinforcement learning signals in the human striatum distinguish learners from nonlearners during reward-based decision making. Journal of Neuroscience 27: 12860-12867.

3. Schwarz GE (1978) Estimating the dimension of a model. Annals of Statistics 6: 461-464.

4. Field A (2009) Discovering statistics using SPSS. London: Sage Publications.

**Supplemental Table S1**. Mean (and SD) of estimated parameter values and model fit (LLE) for the four-parameter model reported in the main text (with α_G_ and α_L,_ *ß*, and *R0* as free parameters), and a five-parameter model that also included a perseveration parameter *P*.

| Model | α_G_ | α_L_ | *ß* | *R0* | *P* | LLE |
| --- | --- | --- | --- | --- | --- | --- |
| α_G_ and α_L,_ *ß*, and *R0* (4 free parameters*)* | 0.27 (0.32) | 0.23 (0.35) | 0.35 (0.27) | 0.35 (0.55) | 0 (fixed) | -81.7 (23.9) |
| α_G_ and α_L,_ *ß*, and *R0,* and *P* (5 free parameters) | 0.21 (0.29) | 0.35 (0.40) | 0.36 (0.26) | 0.26 (0.62) | 0.76 (0.94) | -79.4 (23.9) |
